# Regulation of Osteoclast Differentiation at Multiple Stages by Protein Kinase D Family Kinases

**DOI:** 10.3390/ijms21031056

**Published:** 2020-02-05

**Authors:** Amanda C. Leightner, Carina Mello Guimaraes Meyers, Michael D. Evans, Kim C. Mansky, Rajaram Gopalakrishnan, Eric D. Jensen

**Affiliations:** 1 Department of Diagnostic and Biological Sciences, University of Minnesota School of Dentistry, Minneapolis, MN 55455, USA; 2 Clinical and Translational Science Institute, University of Minnesota, Minneapolis, MN 55455, USA; 3 Department of Developmental and Surgical Sciences, University of Minnesota School of Dentistry, Minneapolis, MN 55455, USA

**Keywords:** osteoclasts, protein kinase D, cellular differentiation, actin cytoskeleton, bone resorption

## Abstract

Balanced osteoclast and osteoblast activity is necessary for skeletal health, whereas unbalanced osteoclast activity causes bone loss in many skeletal conditions. A better understanding of pathways that regulate osteoclast differentiation and activity is necessary for the development of new therapies to better manage bone resorption. The roles of Protein Kinase D (PKD) family of serine/threonine kinases in osteoclasts have not been well characterized. In this study we use immunofluorescence analysis to reveal that PKD2 and PKD3, the isoforms expressed in osteoclasts, are found in the nucleus and cytoplasm, the mitotic spindle and midbody, and in association with the actin belt. We show that PKD inhibitors CRT0066101 and CID755673 inhibit several distinct aspects of osteoclast formation. Treating bone marrow macrophages with lower doses of the PKD inhibitors had little effect on M-CSF + RANKL-dependent induction into committed osteoclast precursors, but inhibited their motility and subsequent differentiation into multinucleated mature osteoclasts, whereas higher doses of the PKD inhibitors induced apoptosis of the preosteoclasts. Treating post-fusion multinucleated osteoclasts with the inhibitors disrupted the osteoclast actin belts and impaired their resorptive activity. In conclusion, these data implicate PKD kinases as positive regulators of osteoclasts, which are essential for multiple distinct processes throughout their formation and function.

## 1. Introduction

Although the rate of bone synthesis by osteoblasts is of high importance to bone growth, maintenance and repair, the process of bone resorption by multinucleated cells highly specialized for breaking down bones called osteoclasts plays an equally important role in skeletal health [1]. Resorption is required to generate marrow cavities and to preserve proper bone geometry during skeletal growth, maintain systemic mineral homeostasis, and is an important component of the fracture repair process. However, tipping the balance between bone formation and bone loss towards greater bone loss drives the skeletal fragility of osteoporosis and other diseases. Osteoclasts can be recruited as agents of bone destruction in cases such as skeletal cancer metastases [2], Paget’s disease of bone [3,4], periodontal disease [5], and rheumatoid arthritis [6]. Bisphosphonates or RANKL (Receptor Activator of NF-kB Ligand) blocking antibodies are used clinically to combat osteolytic diseases. However, these anti-resorptive treatments can lead to adverse effects including hypocalcaemia, atypical femoral fractures, and osteonecrosis of the jaw [7,8,9]. Identification of novel targets influencing bone resorption and development of new therapies is essential to improve treatment options for osteolytic diseases.

Osteoclasts are a unique cell type, notable for being multinucleated syncytia formed by the fusion of mononucleated precursor cells [1,10]. In the most common pathway, osteoclast progenitors are driven to the osteoclast lineage by combined signaling of M-CSF (Macrophage Colony-Stimulating Factor) and RANKL, which promote osteoclast precursor growth and survival, osteoclast lineage specification, and cellular differentiation. One common identifying characteristic of these early preosteoclasts is their expression of tartrate-resistant acid phosphatase (TRAP). These TRAP-positive preosteoclasts become fusion competent in response to various extracellular signals, migrate and adhere together, undergo cytoskeletal rearrangements to bring their plasma membranes into close contact, and finally merge their plasma membranes to generate the syncytial multinucleated osteoclasts [11]. The bone-resorptive capacity of mature osteoclasts is supported by the formation of actin-rich structures called podosomes [12,13,14]. In osteoclasts differentiated on plastic or glass, podosomes transit from a cluster-like organization, through a transient internal ring organization, and ultimately to a large circular structure near the cell periphery called the actin belt or podosome belt [15]. Osteoclasts on bone form a similar-appearing circular structure called the sealing zone. The precise relationship between podosome belts on glass and the sealing zone formed on bone remains controversial [12]. The sealing zone is essential for efficient osteoclast resorptive function by attaching the cell to the underlying bone surface and sealing off the so-called resorptive pit, into which osteoclasts actively secrete acid and proteolytic enzymes to bring about bone erosion. Disruption of the sealing zone perturbs the resorptive process and reduces bone destruction.

Most aspects of cellular biology and protein function are regulated by reversible protein phosphorylation by protein kinases and dephosphorylation by phosphatases. One such group of protein kinases is the Protein Kinase D (PKD) family [16,17]. It consists of three serine/threonine protein kinases, PKD1, PKD2 and PKD3, which share overall high sequence homology to each other. PKD kinases are most closely related to the Ca2+/calmodulin-dependent kinases. They can be recruited to specific subcellular sites by binding to diacylglycerol, and become catalytically activated in response to various stimuli including heterotrimeric G-protein-coupled receptors and receptor tyrosine kinases through phosphorylation of serine 744 and serine 748 residues in the activation loop (using the amino acid numbering of mouse PKD1) [18]. Serine 744 is predominantly phosphorylated by PKC members, while phosphorylation of serine 748 can also be achieved through PKC-independent autophosphorylation [19,20,21]. Activated PKD1 and PKD2 undergo further autophosphorylation of a serine residue near the C-terminus (Ser916 in mouse PKD1); this residue is not conserved in PKD3. This autophosphorylation site is commonly used as an indicator of PKD kinase activity. The PKD proteins are widely expressed and implicated in governing a range of cellular processes including cell migration, gene expression, cell proliferation and death, and subcellular vesicle trafficking [17,22,23].

The function of PKD in osteoclasts is largely unknown. Recent work from our group indicated that osteoclasts mainly express PKD2 and PKD3, and revealed that blocking their function inhibited the differentiation of committed osteoclast precursors prior to the cell–cell fusion step [24]. The goal of the current work is to further characterize the functions of PKDs in osteoclast differentiation and function. Key findings are that in addition to blocking the formation of multinucleated osteoclasts, PKD inhibitors reduce motility and survival of mononucleated preosteoclasts, while in multinucleated mature osteoclasts they disrupt the actin belt and impair resorptive function. Thus, PKD kinases are involved in the regulation of multiple aspects of osteoclast physiology and might represent targets for novel antiresorptive therapies to attenuate pathologic bone loss.

## 2. Results

### 2.1. Determination of PKDs’ Subcellular Localization and Phosphorylation Through Osteoclastogenesis

We previously reported that osteoclasts express Pkd2 and Pkd3 but little or no Pkd1, and showed that drugs that inhibit PKDs in osteoclast cultures yielded TRAP-positive preosteoclasts that were unable to further differentiate into multinucleated, mature osteoclasts [24]. To better understand the roles of PKD proteins in osteoclasts, we used confocal microscopy to examine PKD2 and PKD3 immunofluorescence staining to determine their subcellular localizations throughout differentiation. We used a standard in vitro osteoclast culture system in which non-adherent mouse bone marrow monocytes are stimulated with M-CSF for 48 hours to become adherent M-CSF-dependent BMMs. At this point, which we refer to as Day 0, BMMs are further stimulated with M-CSF and RANKL to become committed TRAP-positive preosteoclasts. Between days 2–3 of M-CSF+RANKL stimulation, these preosteoclasts begin fusing into multinucleated osteoclasts and ultimately mature into large multinucleated osteoclasts by days 4–5. We first performed a preliminary study in which we confirmed the specificity of our PKD2 and PKD3 antibodies. Immunofluorescence experiments in HEK293T cells transfected with mouse Pkd2 or Pkd3 expression plasmids indicated that each antibody specifically recognizes the correct overexpressed protein without evidence of cross reactivity under our conditions (Figure S1A). In addition to looking at total PKD2 and PKD3 proteins, we visualized the pattern of PKD catalytic activation in osteoclasts by staining for phospho-PKD Serine 744/748 (P-Ser744/748), which represents phosphorylation in the kinase activation loop of PKD1, 2 and 3, and for phospho-PKD Serine 916 (P-Ser916), an autophosphorylation site present in PKD1 and PKD2 but not PKD3 that is correlated with catalytic activity.

**Figure 1:**
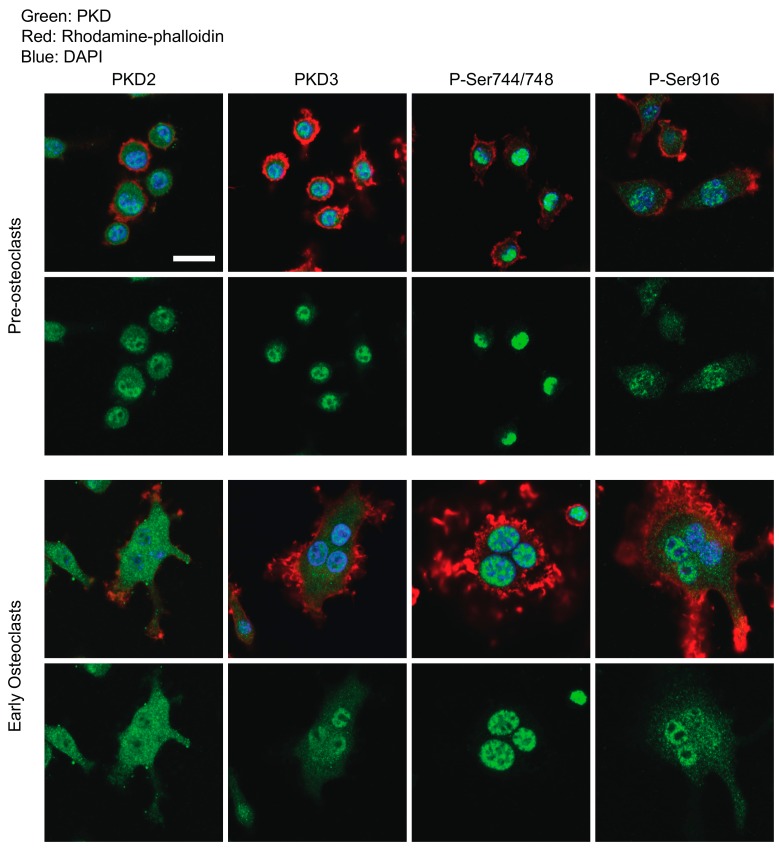
Immunofluorescence localization of PKD2 and PKD3 in pre-osteoclasts and early post-fusion osteoclasts. Osteoclast cultures at preosteoclast and early multinucleated osteoclast stages of differentiation were stained with the indicated PKD antibodies (green), rhodamine-phalloidin (for F-actin, red) and DAPI (for DNA, blue) and imaged by confocal microscopy. Merged images are shown above and the corresponding green channel below. Scale bar = 20 µm. Each of these antibody stainings and timepoints has been performed three independent times with similar results.

Figure 1 upper panels show that in preosteoclasts stained on day 2 after addition of RANKL, total PKD2 and PKD3 are seen largely in the cell nuclei, with smaller amounts of staining in the cytoplasm. P-Ser744/748 and P-Ser916 showed similar staining primarily in nuclei, with a lower amount of cytoplasmic staining. Consistent with previous reports [25], we also observed phospho-PKD staining associated with mitotic spindles (Figure S2A) and as two bright foci at the midbody between cells that appear to be completing cytokinesis (Figure S2B). In immature osteoclast cultures that have recently begun to undergo cell–cell fusion (stained on day 3 RANKL), PKD2 and PKD3 staining was evident in both the cytoplasm and nucleus (Figure 1, lower panels). Both phospho-PKD antibodies showed predominantly nuclear staining with a small amount of diffuse cytoplasmic staining (Figure 1, lower panels).

**Figure 2:**
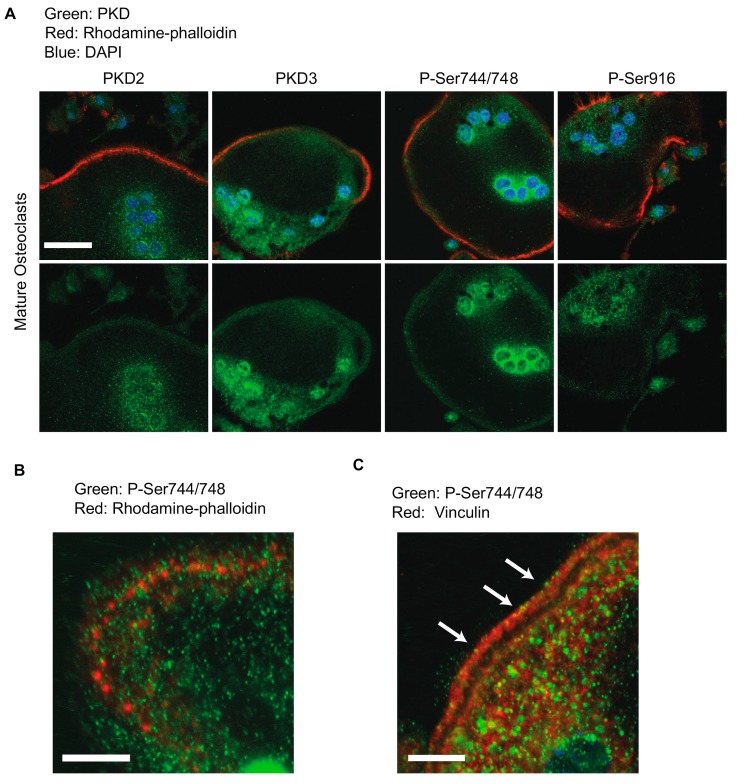
Immunofluorescence localization of PKD2 and PKD3 in mature osteoclasts. (**A**) Mature multinucleated osteoclasts were stained with the indicated PKD antibodies (green), rhodamine-phalloidin (for F-actin, red) and DAPI (for DNA, blue) and imaged by confocal microscopy. Scale bar = 20 µm. (**B**) Higher magnification image showing podosomes assembling into a ring or belt with P-Ser744-positive foci (green) surrounding the F-actin core (red). Scale bar = 10 µm. (**C**) Higher magnification image showing P-Ser744-positive foci (green) co-localizing with vinculin-rich regions (red) adjacent to the actin belt. Arrows highlight examples of these foci. Scale bar = 10 µm. Each of these antibody stainings has been repeated at least three independent times with similar results.

Mature multinucleated osteoclasts displaying well-formed actin belts were fixed and stained 4 days after RANKL treatment (Figure 2A). These cells showed nuclear staining with all four antibodies, although this nuclear staining was somewhat variable in its intensity, and in diffuse cytoplasmic staining surrounding clusters of nuclei. Interestingly, we also noted a band of staining around the periphery of the cells in close proximity to the actin belt (Figure 2A). To further confirm the validity of these staining patterns, we examined negative control slides on which mature osteoclasts were stained without primary antibody but were otherwise imaged and processed identically; no staining on the green channel was detected from these slides (Figure S1B). However, we did observe PKD staining at the periphery of mature osteoclasts that were not stained with rhodamine-phalloidin (Figure S1C). These controls suggest that the PKD staining around the periphery of mature osteoclasts represents real localization to the vicinity of the actin belt. Higher magnification images of the region near the actin belt revealed the presence of P-Ser744/748-stained foci adjacent to the F-actin rich podosome cores (Figure 2B). In the mature actin belt, a dense F-actin ring is flanked on either side by a region containing vinculin and other cytosketal adaptors, integrins and a variety of signaling molecules [14,26]. Shown in Figure 2C, immunostaining against vinculin (red) showed the expected two parallel bands of vinculin flanking an unstained central region that represents the F-actin-dense zone. Foci of P-Ser744/748 (green) were detected within the red domain of vinculin(arrows). Together these data suggest that PKDs are present and active in the nucleus, cytoplasm and actin belt of osteoclasts.

### 2.2. CRT0066101 Inhibits PKD Activity in Osteoclasts

**Figure 3:**
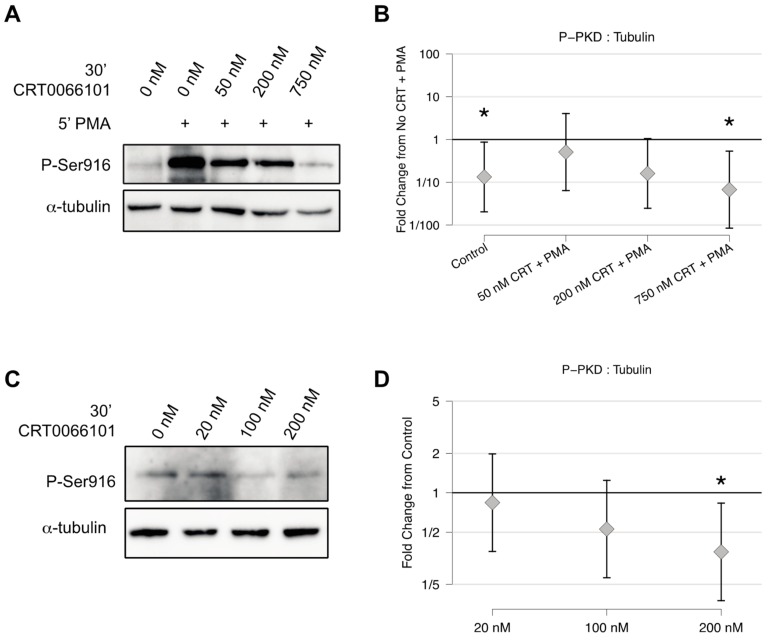
CRT0066101 inhibits PKD activity in osteoclasts. (**A**) Preosteoclasts were pre-treated with increasing doses of CRT0066101 for 30 minutes prior to stimulation with 50 mM phorbol 12-myristate 13-acetate (PMA), indicated by ‘+’ for 5 minutes. (**B**) Quantitation of P-Ser916 levels relative to the level from PMA-alone set as 1.0. * *p* < 0.05 versus PMA alone. (**C**) Treatment of preosteoclasts with the indicated CRT0066101 doses for 30 minutes and blotted against endogenous P-Ser916. (**D**) Quantitation of experiments from (**C**) with the level from untreated cells set as 1.0. * *p* < 0.05 versus control. Each experiment was performed four times. Representative Western blots from single experiments are shown, while the graphs represent the collective results from the independent experiments analyzed together as described in Section 4.12.

Our previous study into PKD function in osteoclasts made use of the PKD inhibitors CID755673 and Gö6976 [24]. Although these inhibitors are effective against PKDs, they are both known to have multiple off-target effects on other kinases. In contrast, no off-target effects have been reported for the newer PKD inhibitor CRT0066101 through testing against a panel of over 90 protein kinases [27] and in its use as an inhibitor of PKD kinases in at least 20 published papers. We thus asked if CRT0066101 inhibits osteoclast differentiation in a similar fashion to CID755673 and Gö6976. To begin, we found that the treatment of preosteoclast cultures with CRT0066101 for 30 minutes dose-dependently reduced the robust induction of P-Ser916 autophosphorylation stimulated by the PKD activator phorbol 12-myristate 13-acetate (PMA) (Figure 3A–B). Similarly, CRT0066101 reduced the much lower levels of endogenous P-Ser916 in a dose-dependent fashion, with strong inhibitory effects seen between 100–200 nM (Figure 3C–D), which is comparable or lower than doses used in similar published works [28,29,30,31]. Collectively these data validate that CRT0066101 effectively inhibits PKD activity in osteoclasts.

### 2.3. CRT0066101 Inhibits in Vitro Osteoclast Differentiation

**Figure 4:**
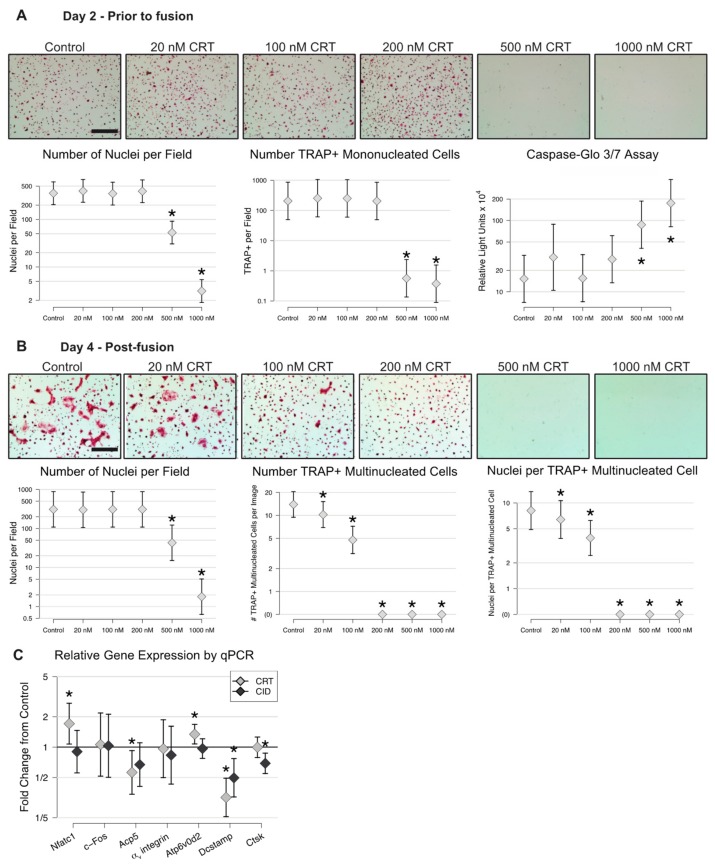
CRT0066101 inhibits the formation of multinucleated osteoclasts. BMMs were stimulated with M-CSF and RANKL in the absence or presence of CRT0066101 for 2 days (**A**) or 4 days (**B**). Cells were fixed, stained for TRAP and DAPI, and counted. The total number of DAPI-positive nuclei, TRAP-positive mononucleated preosteoclasts, TRAP-positive multinucleated osteoclasts, and mean number of nuclei per multinucleated osteoclast are graphed. Scale bars in (**A**) and (**B**) = 300 µm. To measure apoptosis, preosteoclasts were treated with CRT0066101 for 3 hours and assayed using the Caspase-Glo3/7 kit (A—rightmost graph). (**C**) qPCR measurement of the indicated genes in the presence of 200 nM CRT0066101 or 30 µM CID755673. Data are graphed as fold change relative to untreated control (set as 1.0). * *p* < 0.05, versus control.

Our next question was whether CRT0066101 affects osteoclast differentiation in BMM cultures stimulated with M-CSF and RANKL, similarly to what we previously reported for other PKD inhibitors. We began by determining the dose response of osteoclast cultures to CRT0066101. BMMs were stimulated for osteoclast differentiation using M-CSF and RANKL, with treatment of CRT0066101 commencing at day 0 along with the first addition of RANKL. In our first experiment, we treated cells with doses of CRT0066101 between 20 nM and 1000 nM. After 2 days culture, prior to the onset of osteoclast cellular fusion, we stained nuclei with DAPI and counted the number of cells present between groups, and stained the cells with TRAP to detect the induction of preosteoclasts. Although doses of CRT0066101 up to 200 nM had no detected effect on the number of nuclei or the number of cells positive for TRAP, CRT0066101 at 500 nM or 1000 nM dramatically reduced the total number of cells present after 48 hours (Figure 4A photos, left and middle graphs). To determine whether the decreased cell numbers at higher doses were due to apoptosis, we treated BMM cultures for 24 hours with M-CSF and RANKL to induce preosteoclast identity, then stimulated with CRT0066101 for 3 hours, and finally measured activation of Caspases 3 and 7. CRT0066101 up to 200 nM had little effect on Caspase 3/7 activity, while treatments at 500 nM or above produced large and statistically significant increases (Figure 4A, rightmost graph), indicating that higher doses of CRT0066101 do lead to preosteoclast apoptosis. As a further test of whether CRT0066101 affected preosteoclast viability, we treated preosteoclasts with increasing doses of CRT0066101 for 4 or 24 hours, stained with propidium iodide (PI), which is excluded from viable cells but brightly stains nuclei of dead cells, and then photographed them. As a positive control for cell death and PI staining, an additional group of cells was treated with 70% ethanol for 5 minutes immediately before staining. After photographing the PI staining, the cells were fixed, permeabilized and re-stained with DAPI to determine the total number of nuclei present. These experiments are presented in Figure S3. As expected, propidium iodide stained 100% of the cells exposed to 70% ethanol, indicating that they were non-viable. Doses of CRT0066101 up to 200 nM showed no significant effect on the overall cell number or the percent cells PI-positive, either at 4 hours or 24 hours. 500 nM CRT0066101 for 24 hours decreased the number of cells by 45% compared to untreated cultures and increased the absolute number (not shown) and percentage of PI-positive cells from 1% in the untreated group to 5% of the 500 nM group, although this increase did not reach statistical significance. 1000 nM CRT0066101 showed stronger and statistically significant decreases in total cell number, and increases in absolute number (not shown) and percentage PI-positive cells at both timepoints. These data further indicate that doses of CRT0066101 up to 200 nM are not toxic to preosteoclasts while 500–1000 nM gives dose-dependent, progressive reductions to cell viability.

To test whether CRT0066101 impacted the formation of mature osteoclasts, we treated RANKL-stimulated cultures with CRT0066101 from day 0 through day 4. After 4 days, control cultures showed numerous large TRAP-positive multinucleated osteoclasts (Figure 4B). Cultures treated with CRT0066101 between 20 and 200 nM showed no change in the overall number of nuclei present and many TRAP-positive mononucleated cells (Figure 4B photos and left graph), but clear, statistically significant reductions in the number of TRAP-positive multinucleated osteoclasts and the number of nuclei per osteoclast (Figure 4B middle and right graphs), suggesting that CRT0066101 blocked osteoclastogenesis at or shortly before the cell–cell fusion stage. Cultures treated with CRT0066101 at 500 nM and 1000 nM for 4 days showed very few nuclei and did not form multinucleated osteoclasts, which was unsurprising since almost all of the cells had died by day 2. Seeking to further understand the changes in our osteoclast cultures treated with CRT0066101, we performed real-time qPCR on early multinucleated osteoclast cultures similar to those shown in the bottom panels of Figure 1 to measure genes associated with early osteoclast specification and differentiation (Nfatc1 and cFos), osteoclast phenotypic markers (Acp5 and α_v_ integrin and Ctsk), and genes involved with fusion (Atp6v0d2 and Dcstamp). For comparison, we included both untreated controls and cells treated with CID755673, which was previously reported [24], and the data are graphed as fold change relative to untreated controls. These data showed that cultures treated with CRT0066101 or CID755673 showed 50–70% reduction in Dcstamp expression, and 30–40% less Acp5 than controls (Figure 4C). The other genes examined showed either weak or inconsistent changes in expression between the two PKD inhibitors.

### 2.4. CRT0066101 Reduces Preosteoclast Motility

**Figure 5:**
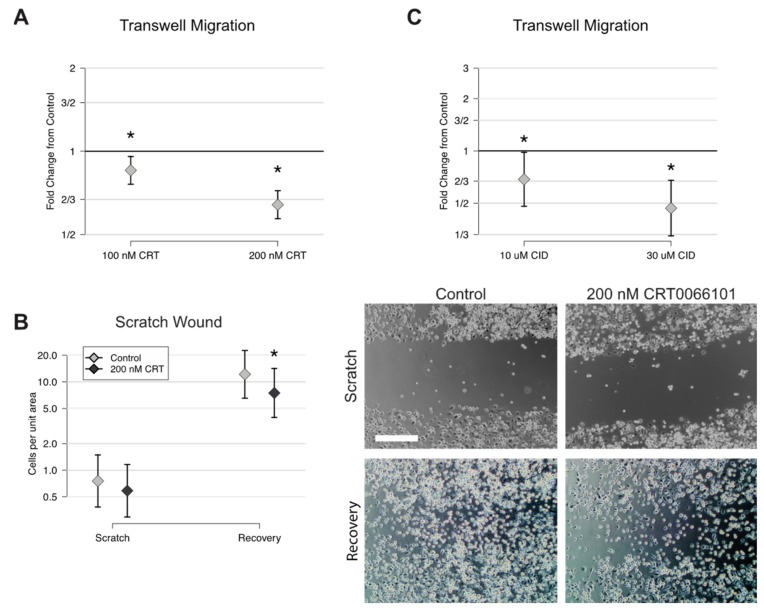
CRT0066101 and CID755673 inhibit preosteoclast cell motility. (**A**) Transwell migration assay showing a proportion of preosteoclasts migrating through the filter in the presence of CRT0066101, graphed relative to control set as 1.0. (**B**) Scratch wound migration assay comparing number of cells present immediately after scratching (Scratch) and 24 hours later (Recovery). Representative photos are shown at right. Scale bar = 300 µm (**C**) Transwell migration assay showing cells treated with CID755673. Each experiment was performed three times with similar results. * *p* < 0.05 versus control.

One of the first steps in the preosteoclast fusion process involves cellular migration to locate partners [11], and since roles for PKD proteins as regulators of cell motility have been described [32], we hypothesized that at least part of the reduction in cell fusion from CRT0066101 might be due to decreased preosteoclast motility. To test this, we performed transwell migration assays in which we asked whether CRT0066101 inhibited the ability of preosteoclasts in serum-free media to migrate through a transwell insert towards complete media containing serum, M-CSF and RANKL. 100 nM CRT0066101 reduced the number of cells migrating through the filter by 15%, while 200 nM CRT0066101 gave 36% reduction in migrated cells on average (Figure 5A). To extend this observation, we performed scratch assays in which densely seeded BMM cultures were scratched with a pipette tip, and the number of cells present in the scratched area was counted immediately afterward and again 24 hours later. We reproducibly observed a 30–40% reduction in the number of cells reentering the scratch area in the presence of 200 nM CRT0066101 (Figure 5B). Consistent with the hypothesis that CRT0066101’s effects on motility are mediated through PKD kinases, we found that 30 µM CID755673 also reduced the number of migrated cells by 55% in the transwell assay compared to the control group, while 10 µM gave a smaller reduction in motility of 31% (Figure 5C). CID755673 was used at 10 and 30 µM concentrations for this experiment based on our previous studies and consistent with the effective dose against PKD activity in other published cell culture studies [24,33,34,35]. Taken together, these data suggest that PKD promotes preosteoclast cellular motility.

### 2.5. CRT0066101 Inhibits Osteoclast Resorptive Activity

**Figure 6:**
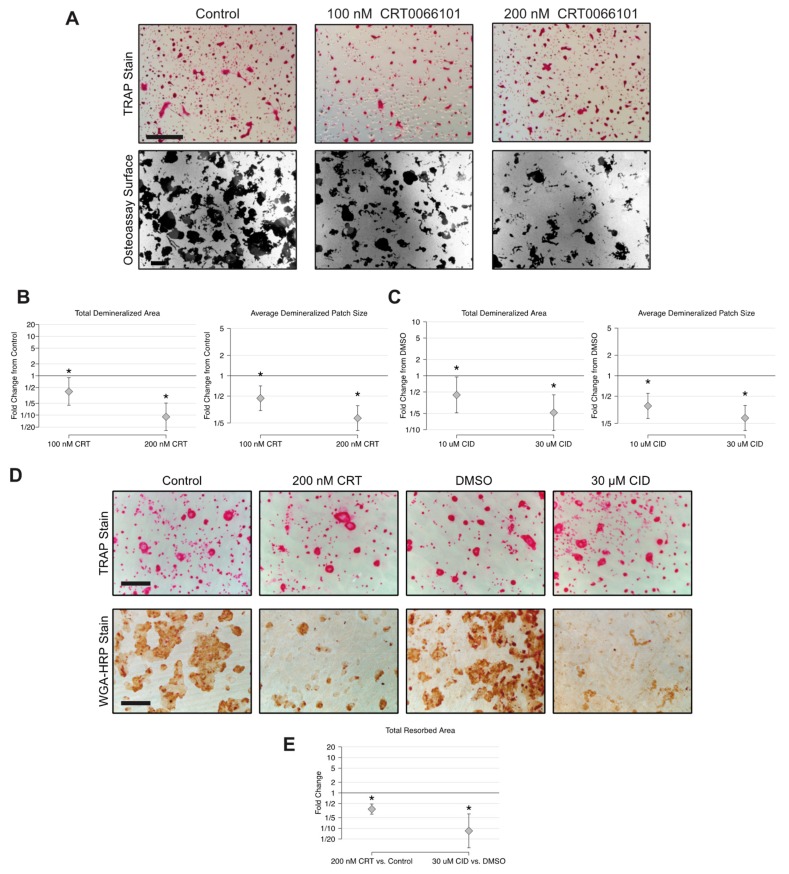
CRT0066101 and CID755673 reduce osteoclast resorptive activity. (**A**) TRAP staining and dark field microscopy of osteoassay plates cultured with mature osteoclasts in the presence of CRT0066101 for 2 days. (**B–C**) Quantitative analysis of average demineralized patch size and total demineralized area relative to the untreated control group (set as 1.0). (**D**) TRAP and wheat germ agglutinin-HRP staining of osteoclasts differentiated on bone slices and then treated with CRT0066101 or CID755673 for 4 days. The experiment was performed three times with similar results. (**E**) Total resorbed area on bone slices relative to the control groups. * *p* < 0.05. Scale bars in A = 300 µm. Scale bar in D = 250 µm.

Since treatment with PKD inhibitors throughout differentiation blocks the formation of multinucleated osteoclasts, the significance of PKDs on the resorptive activity of mature osteoclasts had not yet been tested. To address this, we allowed M-CSF- and RANKL-treated BMMs to differentiate into multinucleated osteoclasts on calcium phosphate-coated osteoassay plates for 4 days and then added 100 or 200 nM CRT0066101 for 2 further days. At the end of the treatment, TRAP staining showed that the overall appearance of osteoclasts was similar between groups (Figure 6A). Imaging the calcium phosphate substrate revealed that the average demineralized patch size and total demineralized area were each strikingly reduced by CRT0066101 in a dose-dependent manner (Figure 6A–B). Additional studies showed that CID755673 also inhibited osteoclast demineralization activity (Figure 6C), lending further support to the hypothesis that osteoclast resorptive activity requires PKD. To verify these findings on authentic bone substrate, we seeded BMMs onto bone slices and cultured until multinucleated osteoclasts had formed. We then cultured for an additional 4 days in the presence of CRT0066101 or CID75563. TRAP staining at the end of the culture period showed no obvious difference in the general appearance of cells on the bone slices, whereas wheat germ agglutinin-HRP staining revealed a significant reduction in the area resorbed in the presence of PKD inhibitors (Figure 6D–E). From these data, we conclude that PKD activity is required for osteoclast bone resorptive activity.

### 2.6. Actin Belt Morphology in Mature Osteoclasts Requires PKD Function

**Figure 7:**
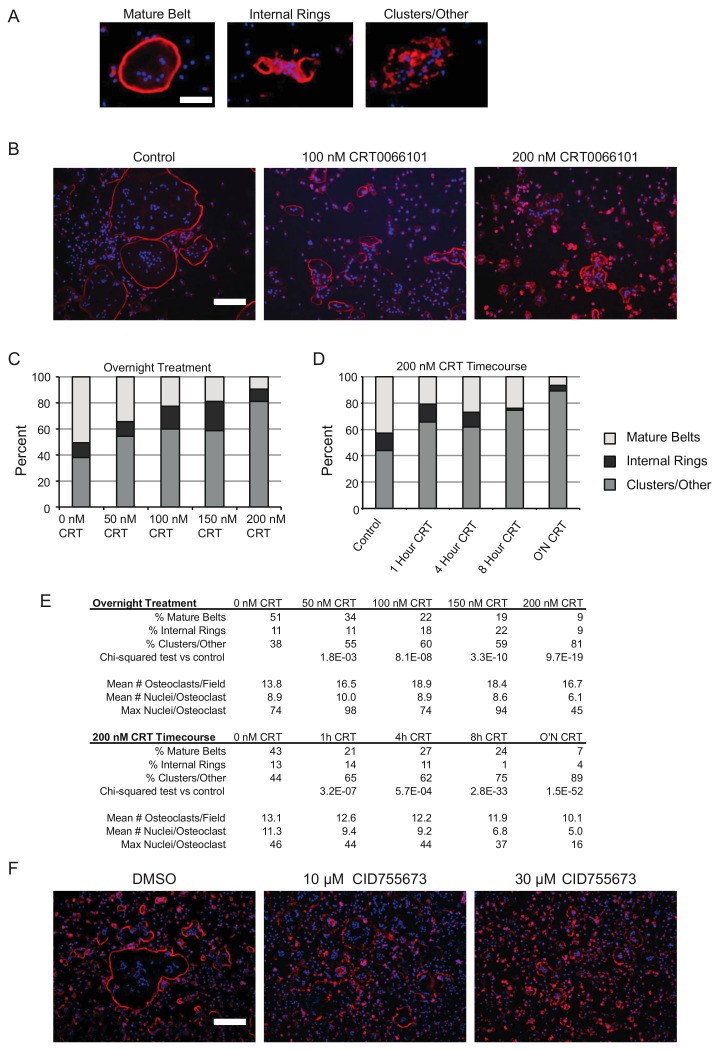
CRT0066101 and CID755673 disrupt the actin ring in mature osteoclasts. (**A**) Typical examples of rhodamine-phalloidin-stained osteoclasts classified as mature actin belt, internal rings, or podosome clusters/other actin cytoskeletal morphologies. Scale bar = 200 µm (**B**) representative photos of rhodamine-phalloidin staining of mature osteoclast cultures treated overnight with CRT0066101. (**C–D**) Relative proportion of each class of actin cytoskeletal morphology after CRT0066101 treatment for the indicated doses and times. Eight random fields containing 150–200 multinucleated cells total per group were analyzed. (**E**) Quantitative analysis of the data presented in (**B**–**D**). (**F**) Rhodamine-phalloidin staining of mature osteoclasts treated overnight with CID755673 or DMSO vehicle. Scale bar = 500 µm.

The mature osteoclast actin cytoskeleton is characterized by the formation of the actin ring or actin belt, depending on the nature of the underlying substrate. These structures seal off an extracellular region highly enriched in acids and proteases to faciliate bone resorption. The formation of these structures is based on self-organization of individual units called podosomes. As described by Destaing et al. [15], podosomes initially form into disorganized groupings or clusters (Figure 7A, right) that will locally assemble into relatively small, transient, circular structures “internal rings” (Figure 7A, middle, not to be confused with bona fide actin rings formed on bone). Continued outward growth of these internal rings finally results in the large, dense actin belt near the periphery of the cells (Figure 7A left). In light of the PKD immunofluorescence staining in the actin belt (Figure 2) and resorption defects (Figure 6), we hypothesized that actin cytoskeletal arrangement in osteoclasts could be disrupted by CRT0066101. To test this, we formed multinucleated osteoclasts on tissue culture plastic, then treated them with CRT0066101 overnight and examined the actin cytoskeleton by staining with rhodamine-phalloidin. While many cells in the control cultures displayed well-organized actin belts, this cytoskeletal architecture was markedly disrupted in the presence of CRT0066101 (Figure 7B,C). Quantitation of these images showed that as CRT0066101 levels increased from zero to 200 nM, the proportion of multinucleated osteoclasts with well-formed actin belts decreased from 51% to 9% of the multinucleated cells, while the proportion of multinucleated cells with podosome clusters or disorganized actin architectures increased from 38% to 81% (Figure 7C). Similar timecourse studies showed progressive disruption of actin belts over time during 200 nM CRT0066101 treatment (Figure 7D,E). Further validating the importance of PKD activity on the actin belt, we treated mature osteoclasts overnight with CID755673, finding a similar disruption of actin belts (Figure 7F). Together these data support the hypothesis that PKDs promote actin belt formation or maintenance, thereby contributing to osteoclastic bone resorption.

## 3. Discussion

The current study represents only the second published work exploring the role of PKD family members in osteoclasts. Our initial work found expression of PKD2 and PKD3 in osteoclasts [24]. *Pkd2* shRNA knockdown, CID755673 and Gö6976 chemical inhibitors were used to show that PKDs promote osteoclast differentiation, with a loss of PKD function arresting differentiation prior to cell–cell fusion. However, the range of PKD effects in osteoclasts was incompletely characterized. We now expand upon the first study, confirming and extending the initial findings using a more specific PKD inhibitor, CRT0066101, determining the cellular sites of PKD localization and activation throughout osteoclast differentiation, and establishing roles in preosteoclast motility, survival, cellular fusion, osteoclast bone resorptive activity, and actin belt dynamics.

The phosphorylation targets and molecular mechanisms of PKDs’ actions in osteoclasts are not yet known although numerous intriguing possibilities for targets of PKD can be identified. A common aspect of cell motility, cell–cell fusion and actin belt maintenance is that each process is highly dependent on careful temporal-spatial regulation of actin cytoskeleton dynamics. These distinct processes are governed by overlapping sets of regulatory proteins. Supporting our observations that PKD localizes to the actin belt, PKD1 has been observed in invasive MDA-MB-231 breast cancer cells localized to invadopodia, actin-rich structures closely related to the podosomes that make up the actin belt [36]. Although the phosphorylation targets of PKD in osteoclasts have not been identified, some important candidate molecules that are involved in the regulation of cell motility or podosomes and are established as phosphorylation substrates for PKD in other contexts include E-cadherin, Cofilin, and Cortactin [see [32] for review].

In addition to their association with actin cytoskeletal elements, we found robust localization of activated PKDs in the nucleus throughout osteoclast differentiation, suggesting that another component of PKD function could involve effects on gene expression. Various molecular mechanisms have been described for PKD’s effects on gene expression including directly phosphorylating transcription factors Snail [37,38,39], FOS/JUN [40,41,42,43], and inhibiting class IIa HDAC transcriptional corepressors [44,45]. PKD has also been linked to effects on p38, p44/42 and JNK pathways [46,47,48], FOS/JUN [40,41,42,43,49,50] and NF-κB signaling [51,52], each of which participates in osteoclast differentiation and represent potential pathways for PKD to influence gene expression during osteoclastogenesis. Since our testing of a small panel of important osteoclast genes only showed reductions to *Acp5*, the gene encoding the key osteoclast marker TRAP, and *Dcstamp*, a key promoter of preosteoclast cell–cell fusion, we suggest that unbiased screening for changes to gene expression could be a more fruitful line of investigation towards determining whether and how PKDs affect gene expression in osteoclasts.

Doses of CRT0066101 up to 200 nM inhibit multinucleated osteoclast formation while appearing to have little effect on preosteoclast proliferation or apoptosis, based on the similar number of nuclei between control cultures and 200 nM CRT0066101. At 500–1000 nM, our data showed dramatic reductions in overall cell number and significant induction of apoptosis. The biological significance of this dose-dependency and the molecular mechanisms responsible remain uncertain. We speculate that the increased cell death from higher doses of CRT0066101 might be connected to the localization of active phospho-PKD to the mitotic spindle and midbody during cytokinesis seen in Supplemental Figure S2 and described by Papazyan [25]. Disruption of the mitotic apparatus leads to cell cycle arrest and ultimately to cell death. Consistent with this idea, a recent publication by Roy and colleagues showed PKD regulation of the G2/M checkpoint, with a loss of PKD2 leading to mitotic catastrophe and apoptosis [53]. Pro-survival mechanisms of PKD action could also involve activation of NF-kB transcription factor [51,52] or the HSP27 cytoprotective chaperone protein [54,55,56].

The possible mechanisms discussed above for the effects of PKD inhibitors on osteoclasts are not exhaustive. PKDs have well known effects on Golgi apparatus function, vesicle trafficking and secretion, cellular proliferation, and other processes [17,22]. Similarly, increasing evidence indicates that each of the PKD proteins can have distinct protein–protein interactions and phosphorylation substrates. The inhibitor-based approach used in this study is expected to block the activity of all three PKD isoforms. Since our data indicate that osteoclasts express PKD2 and PKD3, but not PKD1, better distinguishing the unique or common contributions of these two PKDs to osteoclastogenesis remains an important goal for future work.

In summary, our unique observations establish that PKD is required for osteoclast differentiation and function, indicating these proteins play vital roles as multifaceted regulators of osteoclastogenesis and bone resorption. Since PKD inhibitors reduce osteoclast function, one might propose they could be used as anti-resorptive therapies. However, due to PKDs’ wide tissue distribution and other known physiological roles, we suggest that potential skeletal benefits of long term administration of PKD inhibitors as anti-resorptives may be countered by negative effects on osteoblastic bone formation [57,58], or on other tissues including pancratic β-cells, skeletal muscle, and B- and T-cells; see [59] for a review. Rather, we suggest either that if osteoclast-targeted delivery can be developed or if osteoclast-specific PKD targets can be identified, these might be more viable loci for novel clinical interventions.

Continued knowledge gaps around upstream activation and the downstream targets of PKD and the roles of the individual PKD family members indicate the need for future investigations to reveal novel regulatory pathways involved in skeletal homeostasis, health and disease.

## 4. Materials and Methods

### 4.1. Ethics Statement

These studies were performed in accordance with approval from the University of Minnesota IACUC (Protocol Number 1710-35270A, approved 20 Nov 2017).

### 4.2. Mice and in Vitro Osteoclast Culture

C57BL/6 mice (Jackson Laboratory, Bar Harbor, ME, USA), were housed at the University of Minnesota Research Animal Resources facility according to NIH and IACUC guidelines. Experiments were performed with cells isolated from either male or female mice with similar results; individual experiments only compared cells of a single sex. Osteoclast culture was performed as described [60]. Mice at 2–4 months age were euthanized by CO_2_ asphyxiation and dissected to isolate the femurs and tibiae. Marrow was flushed from the bones, treated with red blood cell lysis buffer (150 mM ammonium chloride, 10 mM potassium bicarbonate, 0.1 mM EDTA pH 7.4) and the isolated cells cultured overnight in αMEM supplemented with 5% FBS, 1% penicillin-streptomycin and 1% CMG14-12 cell supernatant as a source of M-CSF [61]. The following day, the nonadherent population was re-seeded to tissue culture plates at 100,000 cells per cm^2^ and incubated for 48 hours to generate bone marrow macrophages (BMMs), a timepoint we will refer to as Day 0. Cells were then stimulated with 1% CMG14-12 supernatant and 20 ng/mL RANKL (R&D Systems) for up to 5 days to generate mature osteoclasts, with fresh media added every 2 days. Where indicated, cells were treated with PKD inhibitors CRT0066101 (Tocris, Minneapolis, MN, USA) dissolved in sterile PBS, CID755673 (Tocris) in DMSO, or an equal volume of the corresponding PBS or DMSO vehicle controls. For experiments with CID755673, the final DMSO concentration was 0.05% in both the treated and the control groups.

### 4.3. Immunofluorescence Microscopy

Osteoclasts were cultured on glass coverslips for the indicated times. Cells were washed with PBS and fixed with 4% formaldehyde in PBS for 10 minutes. They were then permeabilized with PBS, 0.3% Triton X-100 for 5 minutes, blocked for 30 minutes in blocking buffer: PBS, 0.1% Triton X-100, 3% BSA. Cells were incubated with primary antibodies in blocking buffer overnight at 4 °C, washed three times for 5 minutes with PBS, 0.1% Triton X-100, then incubated with secondary antibodies and rhodamine-phalloidin at 0.2 units/mL (Thermo Fisher, Waltham, MA, USA) for 2 hours. After three more washes, they were mounted in SlowFade Diamond with DAPI (Thermo Fisher) and examined using an Olympus Fluoview 500 laser scanning confocal microscope. Images were acquired at 60× magnification using sequential scanning mode to minimize crosstalk between channels. Staining and confocal microscopy imaging of each antibody/timepoint has been performed independently three times with similar results.

Antibodies used were: PKD2 (Abcam, Cambridge, MA, USA, Ab51250) at 1:50 dilution, PKD3 (Bethyl Laboratories, Montgomery, TX, USA, A300-319A) at 1:30 dilution, Phospho-PKD Ser916 (Cell Signaling Technology, Danvers, MA, USA, #2051) at 1:25 dilution, Phospho-PKD Ser744/748 (Cell Signaling Technology #2054) at 1:40 dilution, Vinculin (Millipore Sigma, St. Louis, MA, USA, V4505) at 1:100, Goat anti-Rabbit AlexaFluor 488 (Thermo Fisher A-11008) at 1:700 dilution, and Goat-antiMouse-AlexaFluor 555 (Thermo Fisher A-21424) at 1:700 dilution. Preliminary studies using FLAG-tagged mouse PKD2 and PKD3 proteins overexpressed in HEK293T cells indicated that these PKD2 and PKD3 antibodies specifically stained only the correct PKD protein under these conditions (Figure S1A).

For assessment of the actin belt morphology presented in Figure 7, osteoclasts on tissue culture dishes were fixed, permeabilized, and stained for rhodamine-phalloidin and DAPI as above. Images were captured at 10× magnification on an Olympus IX70 inverted microscope equipped with a DP72 digital camera. Images were processed with Adobe Photoshop. Quantitation of nuclei number and actin belt morphology were determined manually with the investigator blinded to the treatment group. Eight random fields containing 150–200 multinucleated cells total per group were analyzed. Each experiment was repeated independently three times with similar results.

### 4.4. Western Blotting

Pre-fusion osteoclasts were washed with PBS then lysed in ice cold Modified RIPA Buffer supplemented with Halt Protease and Phosphatase Inhibitor Cocktail (Thermo Fisher). Proteins were resolved on 8% SDS-PAGE and transferred to Immobilon-P PVDF Membrane (Thermo Fisher). Blots were blocked and incubated with primary antibodies diluted at 1:1000 in TBST, 3% BSA at 4 °C overnight. They were washed three times for 5 minutes with TBST, then incubated for 2 hours with HRP-conjugated secondary antibodies at 1:6000 dilution in TBST, 5% nonfat milk, and visualized using WesternBright Sirius chemiluminescent substrate (Advansta, San Jose, CA, USA). Antibodies used were the same PKD antibodies as for immunofluorescence or α-tubulin (Cell Signaling Technology #2144). Band quantitation was performed using Image Lab 6.0 Software (Bio-Rad, Hercules, CA, USA). P-PKD intensities were normalized to α-tubulin from the same lane, and then graphed as fold change relative to the indicated comparison group. Each of the experiments shown in Figure 3 was performed and quantitated four independent times with comparable results. Representative Western blot images from a single experiment are shown, while the accompanying graphs show the aggregate data from all four of the experiments performed. Please refer to Section 4.12 for details on quantitative data analysis.

### 4.5. TRAP Staining

For TRAP staining osteoclast cultures, cells were washed with PBS, fixed with 4% formaldehyde in PBS for 10 minutes then incubated with prewarmed TRAP staining solution (50 mM sodium acetate buffer pH 5.0, 0.1% Triton X-100, 30 mM sodium tartrate, 100 µg/mL napthol AS-MX, 3 µg/mL fast red violet LB) at 37 °C for 5–10 minutes until stained sufficiently. Color development was stopped by washing twice with PBS. Cells were then counterstained with DAPI. The experiments shown in Figure 4A–B were performed independently three times with similar results. Each experiment consisted of four wells per group. For quantitation, three random fields were photographed for DAPI and TRAP from each of the four wells using an Olympus BX-51 microscope equipped with Olympus DP71 digital camera, and the number of DAPI-stained nuclei and TRAP-positive cells determined using NIH ImageJ. TRAP-positive cells containing three or more nuclei were classified as osteoclasts.

### 4.6. Caspase 3/7 Assay

Osteoclast cultures were treated with CRT0066101 at indicated doses for 3 hours. Caspase-Glo 3/7 Assay Reagent (Promega, Madison, WI, USA) was added to cells for 90 minutes at room temperature and read on a GloMax 20/20 Luminometer (Promega) according to the manufacturer’s recommendations. The average background value was subtracted prior to analysis. This experiment was performed three independent times with four replicates per treatment group, giving similar results each time.

### 4.7. Toxicity Assay

Preosteoclasts were treated with the indicated doses of CRT0066101 for either 4 or 24 hours. At the end of the treatment period, cultures were washed twice with PBS and then stained with propidium iodide (PI, Thermo Fisher) at 1µg/mL. Three random fields were immediately photographed at 10× magnification on an Olympus IX70 microscope. After the PI-stained cells were photographed they were fixed with 4% formaldehyde, permeabilized with 0.1% triton X-100 in PBS, stained for DAPI, and re-photographed. Positively stained cells were counted using NIH ImageJ.

### 4.8. Quantitative RT-PCR

Osteoclast cultures were differentiated for 4 days with M-CSF and RANKL with the addition of 200 nM CRT0066101 or 30 µM CID755673 beginning at the first addition of RANKL at Day 0. RNA was isolated using Trizol Reagent (Thermo Fisher), reverse transcribed using iScript cDNA Synthesis Kit (BioRad) and subjected to qPCR amplification using iTaq Sybr Green Supermix (BioRad) on a CFX Connect Real Time PCR System (BioRad). Primer sequences used were: *Hprt1* for 5’gaggagtcctgttgatgttgccag, rev 5’ggctggcctataggctcatagtgc; *Nfatc1* for 5’tcatcctgtccaacaccaaa, rev 5’tcaccctggtgttcttcctc; *cFos* for 5’ccaagcggagacagatcaactt, rev 5’tccagtttttccttctctttcagcaga; *Apc5* for cgtctctgcacagattgca, rev gagttgccacacagcatcac; *Itgav* (αv-integrin) for 5’cctcagagagggagatgttcacac, rev 5’aactgccaagatgatcacccacac; *Dcstamp* for 5’gggcaccagtattttcctga, rev 5’tggcaggatccagtaaaagg; *Atp6v0d2* for 5’tcagatctcttcaaggctgtgctg, rev 5’gtgccaaatgagttcagagtgatg; *Ctsk* for 5’agggaagcaagcactggatc, rev 5’gctggctggaatcacatctt. For each sample, target gene expression was normalized to Hprt1. The resulting normalized gene expression values are graphed as fold change relative to untreated control groups. This experiment was performed with similar results three independent times using triplicate samples for each treatment group. Marker expression levels from qPCR were compared between treatment conditions (control, CRT, CID) using linear mixed-effects models with fixed effects for condition and random effects for experiment. Analyses were performed on the log scale for approximate normality and homogeneity of variance, and then exponentiated to report as ratio changes. Results are reported as estimated marginal means with 95% confidence intervals; confidence intervals are adjusted for multiple comparisons using the Dunnett method for comparing multiple treatment conditions to a control condition. Treatments with adjusted 95% CIs that do not include 1 (on the fold change scale) are regarded as statistically significant.

### 4.9. Transwell Migration Assay

BMMS were cultured in the presence of M-CSF and RANKL for 24 hours. The cells were then scraped, collected, and plated onto Transwell chambers with 8.0 µm polycarbonate membranes (Corning, Corning NY, USA). Cells were seeded into the upper chamber in serum free media plus CRT0066101, CID755673 or vehicle. The lower chamber contained complete osteoclast culture media with 5% FBS, 1% CMG14-12 supernatant, 20 ng/mL RANKL and CRT0066101 or CID755673. The cells were allowed to migrate for 16 hours. Any cells remaining on the upper surface of the membrane were removed with a cotton swab. Cells that migrated through the membrane were fixed with 4% formaldehyde, stained, photographed on an Olympus IX70 microscope with DP71 digital camera, and counted using NIH ImageJ. Experiments were performed three independent times with 3–4 wells per group with similar results.

### 4.10. Scratch Wound Migration Assay

BMM cultures were grown with RANKL and M-CSF for 2 days. The cell layer was scratched with a p200 pipette tip and photographed. The cells were then cultured for an additional 24 hours and re-photographed. The bottom surface of the tissue culture plates was marked with reference lines to enable re-visualization of the same area at both timepoints. Two fields were photographed with an Olympus IX70 microscope with DP71 digital camera for each well immediately after scratching and again after the recovery period. The number of cells in the scratched area was manually counted with the investigator blinded to treatment groups. This experiment was performed three times with three wells per group.

### 4.11. Resorption Assays

For demineralization assays, BMMs were cultured under osteoclastogenic culture as above on OsteoAssay Plates (Corning) until the appearance of large multinucleated osteoclasts. CRT0066101, CID755673 or a vehicle was added, and the cultures were incubated for a further 2 days. Cells were removed by treatment with 5% bleach, rinsed with water, and air dried. Demineralized areas were visualized using darkfield microscopy using an Olympus IX70 microscope with Olympus DP71 digital camera and analyzed using NIH ImageJ. Demineralization assays were performed by four independent experiments for CRT0066101 and three experiments for CID755673, with 3–4 replicates per group.

For resorption assays on bone slices, BMMs were seeded onto bone slices (Immunodiagnostic Systems, Tyne & Wear, UK) and cultured in standard osteoclast differentiation conditions as described in Section 2.2 for 4 days, at which point many large multinucleated osteoclasts were present on the bone slices. At that time, the cultures were switched to αMEM media at pH 6.8 (containing M-CSF and RANKL) to promote their resorptive activity [62] and treated with 200 nM CRT0066101, 30 µM CID755673 or control for 4 additional days. At the end of the culture period, osteoclasts were TRAP stained and photographed. The cells were then removed from the bone slices with a cotton swab. To stain resorbed regions, the slices were incubated with 20 µg/mL HRP-conjugated wheat germ agglutinin (Biotium, Fremont, CA, USA) in PBS for 30 minutes, washed three times, and developed using DAB substrate kit (Biotium). Resorbed areas were quantified using NIH ImageJ. This experiment was performed three times with triplicate samples per group.

### 4.12. Experimental Replication and Statistical Analysis

As indicated throughout the Materials & Methods section, each experiment was performed independently at least three times. Experiments were performed with either three or four replicates per group. Representative photographs of Western blot experiments from within single experiments are shown. For quantitative analysis, all of the data from the independent experiments were pooled together. Comparison between treatment groups was achieved using linear mixed-effects models with fixed effects for condition and random effects for experiment. Analyses were performed on the log scale for approximate normality and homogeneity of variance, then exponentiated to report on the original scale. Results are reported as estimated marginal geometric means with 95% confidence intervals. For experiments with a hierarchical design, e.g. multiple wells per condition and multiple observations per well, a nested random effects structure was used in the model to reflect this design. Count data were analyzed using generalized linear mixed-effects models with the negative binomial family. *p*-values and confidence intervals are adjusted for multiple comparisons using the Dunnett method for comparing multiple treatment conditions to a control condition. An adjusted p-value less than 0.05 is regarded as statistically significant. In Figure 7, differences in actin cytoskeleton morphology between cell populations were tested using the Chi-squared test.

## Supplementary Materials

Supplementary materials can be found at https://www.mdpi.com/1422-0067/21/3/1056/s1.

## Abbreviations

RANKL	Receptor Activator of NF-kB Ligand
M-CSF	Macrophage Colony Stimulating Factor
TRAP	Tartrate-Resistant Acid Phosphatase
PKD	Protein Kinase D
BMM	Bone Marrow Macrophage
PMA	Phorbol 12-myristate 13-acetate
