# Supplementary material for: Regulation of Osteoclast Differentiation at Multiple Stages by Protein Kinase D Family Kinases
Source: Int J Mol Sci. 2020 Feb 5;21(3):1056. doi: 10.3390/ijms21031056 (PMC7036879; doi:10.3390/ijms21031056)
Supplement: Supplementary file 1 [file ijms-21-01056-s001.pdf]

Article

Regulation of Osteoclast Differentiation at Multiple Stages by Protein Kinase D Family Kinases

Amanda C. Leightner [1], Carina Mello Guimaraes Meyers [1], Michael D. Evans [2], Kim C. Mansky [3], Rajaram Gopalakrishnan [1] and Eric D. Jensen [1,*]

[1] Department of Diagnostic and Biological Sciences, University of Minnesota School of Dentistry, Minneapolis, Minnesota 55455, USA; leightner.amanda@gmail.com (A.C.L), carinamgs@yahoo.com.br (C.M.G.M), gopal007@umn.edu (R.G.), jens0709@umn.edu (E.D.J)

[2] Clinical and Translational Science Institute, University of Minnesota, Minneapolis, Minnesota 55455, USA; evan0262@umn.edu

[3] Department of Developmental and Surgical Sciences, University of Minnesota School of Dentistry, Minneapolis, Minnesota 55455, USA; kmansky@umn.edu

* Correspondence: jens0709@umn.edu; Tel.: +1-612-626-4159

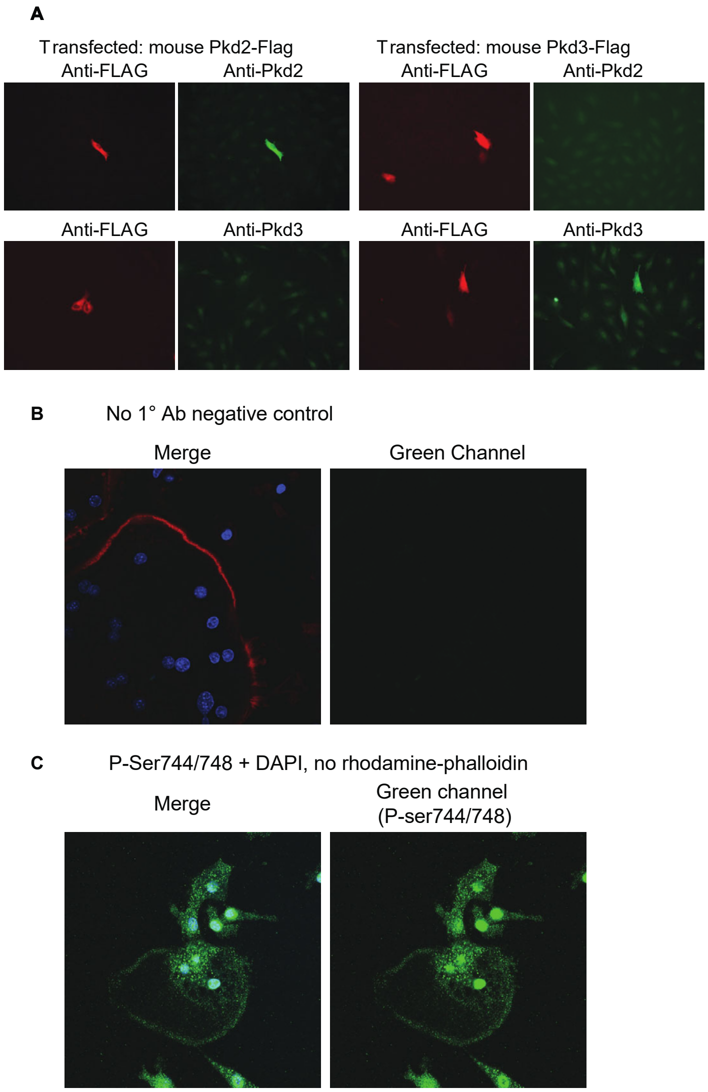

Supplemental Figure S1. Immunofluorescence imaging controls (**a**) Validation of PKD antibody specificity. HEK293T cells were transfected with plasmids expressing FLAG-tagged PKD2 or PKD3 and stained with mouse anti-FLAG (red) and rabbit anti-PKD (green) antibodies as indicated (**b**) Negative control immunofluorescence staining of a multinucleated osteoclast stained with no primary antibody, then anti-rabbit-Alexa488 secondary antibody (green) plus rhodamine-phalloidin (red) and DAPI (blue) (**c**) multinucleated osteoclast stained with anti-P-Ser744/748 and DAPI, but no rhodamine-phalloidin. Panels (b-c) were stained, imaged and photographed by confocal microscopy and processed identically to images shown in Figure 2.

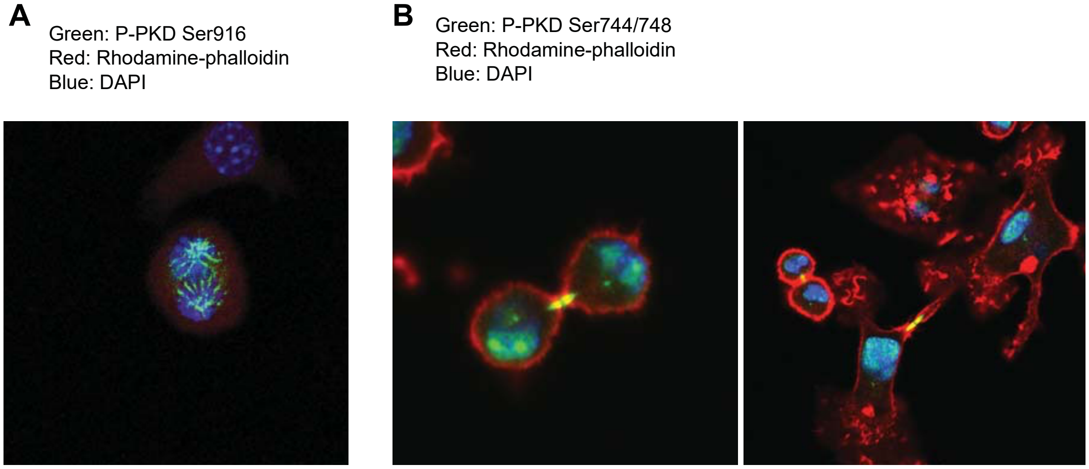

Supplemental Figure S2. PKD localization in mitosis (**a**) P-PKD Ser916 localization during mitosis. Preosteoclasts stained with P-Ser916 (green) showing association with the mitotic spindle. (**b**) P-Ser744/748 (green) showing phosphorylated PKD localization to the midbody. Cells were counterstained with rhodamine-phalloidin (red) and DAPI (blue).

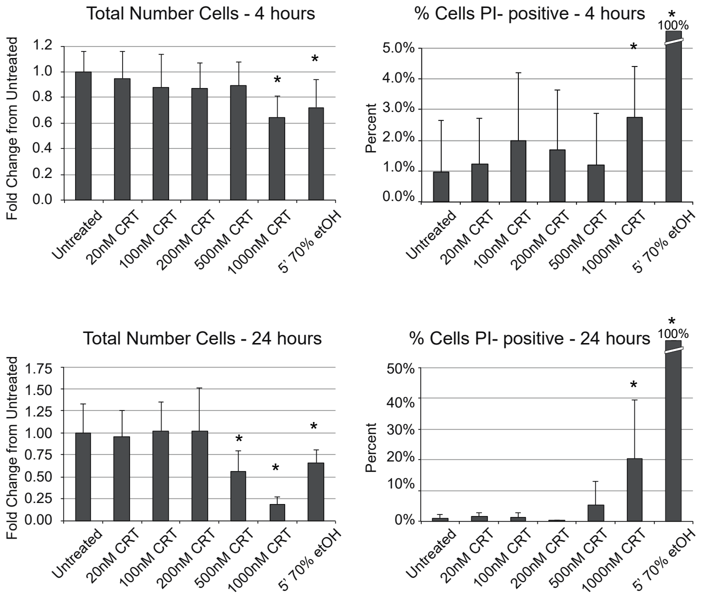

Supplemental Figure S3. CRT0066101 Toxicity Assay. Preosteoclasts were treated with the indicated doses of CRT0066101 for 4 or 24 hours, or with 70% ethanol for five minutes immediately before staining. Non-viable cells were stained by propidium iodide. For total cell number, cells were fixed with formaldehyde then stained with DAPI. Data shown are aggregated data from three experiments that each showed similar results. Note that 100% of the cells in the 70% ethanol group were PI-positive. * $p<0.05$ versus untreated cells.
